# Natural killer group 2D receptor and its ligands in cancer immune escape

**DOI:** 10.1186/s12943-019-0956-8

**Published:** 2019-02-27

**Authors:** Shixin Duan, Weihua Guo, Zuxing Xu, Yunbo He, Chuting Liang, Yongzhen Mo, Yian Wang, Fang Xiong, Can Guo, Yong Li, Xiaoling Li, Guiyuan Li, Zhaoyang Zeng, Wei Xiong, Fuyan Wang

**Affiliations:** 1 NHC Key Laboratory of Carcinogenesis (Central South University) and Hunan Key Laboratory of Translational Radiation Oncology, Hunan Cancer Hospital and the Affiliated Cancer Hospital of Xiangya School of Medicine, Central South University, Changsha, Hunan China; 2 The Key Laboratory of Carcinogenesis and Cancer Invasion of the Chinese Ministry of Education, Cancer Research Institute, Central South University, Changsha, Hunan China; 3 Hunan Key Laboratory of Nonresolving Inflammation and Cancer, Disease Genome Research Center, the Third Xiangya Hospital, Central South University, Changsha, Hunan China; 4 Department of Cancer Biology, Lerner Research Institute, Cleveland Clinic, Cleveland, OH USA; 5 Department of Immunology, School of Basic Medical Science, Central South University, Changsha, Hunan China

**Keywords:** Tumor, Tumor escape, Tumor microenvironment, Natural killer cell, Natural killer group 2D ligand, Natural killer group 2D receptor

## Abstract

The immune system plays important roles in tumor development. According to the immune-editing theory, immune escape is the key to tumor survival, and exploring the mechanisms of tumor immune escape can provide a new basis for the treatment of tumors. In this review, we describe the mechanisms of natural killer group 2D (NKG2D) receptor and NKG2D ligand (NKG2DL) in tumor immune responses.

Natural killer (NK) cells are important cytotoxic cells in the immune system, and the activated NKG2D receptor on the NK cell surface can bind to NKG2DL expressed in tumor cells, enabling NK cells to activate and kill tumor cells. However, tumors can escape the immune clearance mediated by NKG2D receptor/NKG2DL through various mechanisms. The expression of NKG2D receptor on NK cells can be regulated by cells, molecules, and hypoxia in the tumor microenvironment. Tumor cells regulate the expression of NKG2DL at the level of transcription, translation, and post-translation and thereby escape recognition by NK cells. In particular, viruses and hormones have special mechanisms to affect the expression of NKG2D receptor and NKG2DL. Therefore**,** NKG2D\NKG2DL may have applications as targets for more effective antitumor therapy.

## Background

The immune surveillance function of the immune system has been a hot topic in cancer research in recent years. According to the theory of immune surveillance, the body’s immune system functions in surveillance and can kill malignant cells over time. Clinical data have shown that immune surveillance plays an important role in inhibiting and killing tumor cells in many types of human cancers, such as colon cancer and breast cancer [1]. In addition, mouse cancer models have confirmed the existence of “immune editing,” which includes three phases: elimination, equilibrium, and escape. The immune escape phase is the key to tumor cell survival and involves several mechanisms, including reducing the recognition and stimulation of immune cells through tumor cell antigen deletion or modulation, costimulus signal abnormalities, and antigen presentation abnormalities; enhancing expression or secretion of immunosuppressive molecules by tumor cells; and inducing regulator T cells (Tregs) and myeloid-derived suppressor cells (MDSCs) by tumor cells [2]. Exploring the mechanisms of tumor escape is essential for identification of novel therapeutic targets.

Natural killer (NK) cells are important cytotoxic cells of the immune system and participate in innate and adaptive immunity. NK cells play important roles in tumor escape mechanisms. NK cells exert functions in immune surveillance owing to their inhibitory and activating receptors on the surface of the cell membrane. Under normal conditions, NK cells are in an inhibitory state. When activating receptors are activated, NK cells exert cytotoxic effects and kill tumor cells. Animal experiments have shown that mice with underdeveloped or defective NK cells have higher cancer rates [1]. Moreover, clinical data have shown that breast cancer, pancreatic cancer, and prostate cancer [3–5] all lead to poor prognosis by evading the immune surveillance of NK cells.

Among the activating receptors on NK cells, natural killer group 2 member D (NKG2D) receptor is expressed in NK cells as well as many T cells, such as NKT cells, CD8+ T cells, and γδT cells. However, in T cells, NKG2D usually acts only as a costimulatory receptor and does not directly mediate cytotoxicity, which is different from NK cells. NKG2D ligand (NKG2DL), which is often expressed in tumor cells, can activate NK cells by binding to NKG2D receptor, and activated NK cells can then kill tumor cells. When combined with NKG2D receptor on T cells, costimulatory signals can be provided. However, there are various mechanisms that inhibit the action of NKG2D receptor\NKG2DL to enable immune escape of tumor cells. Indeed, experiments have shown that the percentages of NKG2D-positive NK cells in pancreatic, gastric, and colorectal cancers are decreased, and this decrease is associated with poor prognosis in these malignant tumors [6].

Therefore, in this review, we discuss how expression of the NKG2D receptor and its ligands are regulated to enable tumor immune escape in order to provide theoretical basis for clinical treatment.

### Function of NKG2D receptor and its ligands

In NK cells, there are two major types of receptors expressed to control the balance between the activation and inhibition of NK cells: one to identify MHC I molecules, and one to identify non-MHC I molecules. The former includes killer lectin-like receptor (KLR) formed by combination of CD94 with either NKG2A or NKG2C and killer immunoglobulin-like receptor (KIR) [7], and the latter includes NKG2D and natural cytotoxicity receptor (NCR). KIRs identify MHC class I molecules (HLA-A, HLA-B, HLA-C) [8], which have two subtypes, KIR2D and KIR3D and can be divided into KIR2DL, KIR3DL, KIR2DS, and KIR3DS according to the length of the amino acid sequence in the cytoplasmic region. KIR2DL and KIR3DL contain immunoreceptor tyrosine-based inhibitory motif (ITIM) sequences that inhibit NK cells, and KIR2DS and KIR3DS combine with immunoreceptor tyrosine-based activation motif (ITAM)-containing DNAX activating protein 12 (DAP12) to activate NK cells [9, 10]. NKG2A contains an ITIM sequence, which can inhibit NK cells, whereas NKG2C combines with DAP12 to activate NK cells. Both identify nonclassical MHC I molecules (HLA-E) [11]. NCRs include NKp46, NKp30, and NKp44; however, the ligands identified by these molecules have not yet been identified. NKp46 and NKp30 bind to CD33, which contains an ITAM, and NKp44 binds to DAP12, both of which can activate NK cells [12] (Table 1).

**Table 1:** Activation and inhibition receptors of NK cells

Receptor	Ligand	Function	Reference
NKG2A/CD94	HLA-E	Inhibition	[11]
NKG2C/CD94	HLA-E	Activation	[11]
KIR2DL	HLA-A, HLA-B, HLA-C	Inhibition	[8–10]
KIR2DS	HLA-A, HLA-B, HLA-C	Activation	[8–10]
KIR3DL	HLA-A, HLA-B, HLA-C	Inhibition	[8–10]
KIR3DS	HLA-A, HLA-B, HLA-C	Activation	[8–10]
NKG2D	MICA\B, ULBP1–6	Activation	[21]
NKp30	n.d	Activation	[12]
NKp44	n.d	Activation	[12]
NKp46	n.d	Activation	[12]

*n.d* not determined

NKG2D receptor is a homodimer containing two type II transmembrane glycoproteins with a C-type lectin-like structure outside the cell membrane. Human NKG2D receptor is encoded by the killer cell lectin-like receptor subfamily K, member 1 gene and is located in the NK gene complex of chromosome 12, i.e., chromosome 12p13.2. NKG2D may be mistaken for having functions similar to those of members of the NKG2 family; however, this protein has low homology with NKG2A and NKG2C. NKG2D has two different isoforms generated by alternative splicing: the short isoform NKG2D-S and the long isoform NKG2D-L [13]. NKG2D-S is able to combine with both DNAX activating protein 10 (DAP10) and DAP12, whereas NKG2D-L only binds to DAP10. DAP10 has a YXXM (Tyr.X.X-Meth) sequence in the cytoplasm of the cell, which functions to recruit phosphatidylinositol 3-kinase (PI3K) and growth factor receptor bound protein 2 (GRB2) [14] to induce the cytotoxicity and survival of cells [15]. DAP12 has an ITAM, which functions to recruit spleen tyrosine kinase (Syk) and Zeta-chain-associated protein kinase 70 (ZAP70) to induce cytotoxicity and cytokine release [16]. In mice, immune cells express both the NKG2D-L and NKG2D-S subtypes. Thus, murine NKG2D can bind to both DAP10 and DAP12 [2]. Humans only express the NKG2D-L subtype; accordingly, human NKG2D receptor can only bind to DAP10 to form the NKG2D complex [17].

**Fig. 1:**
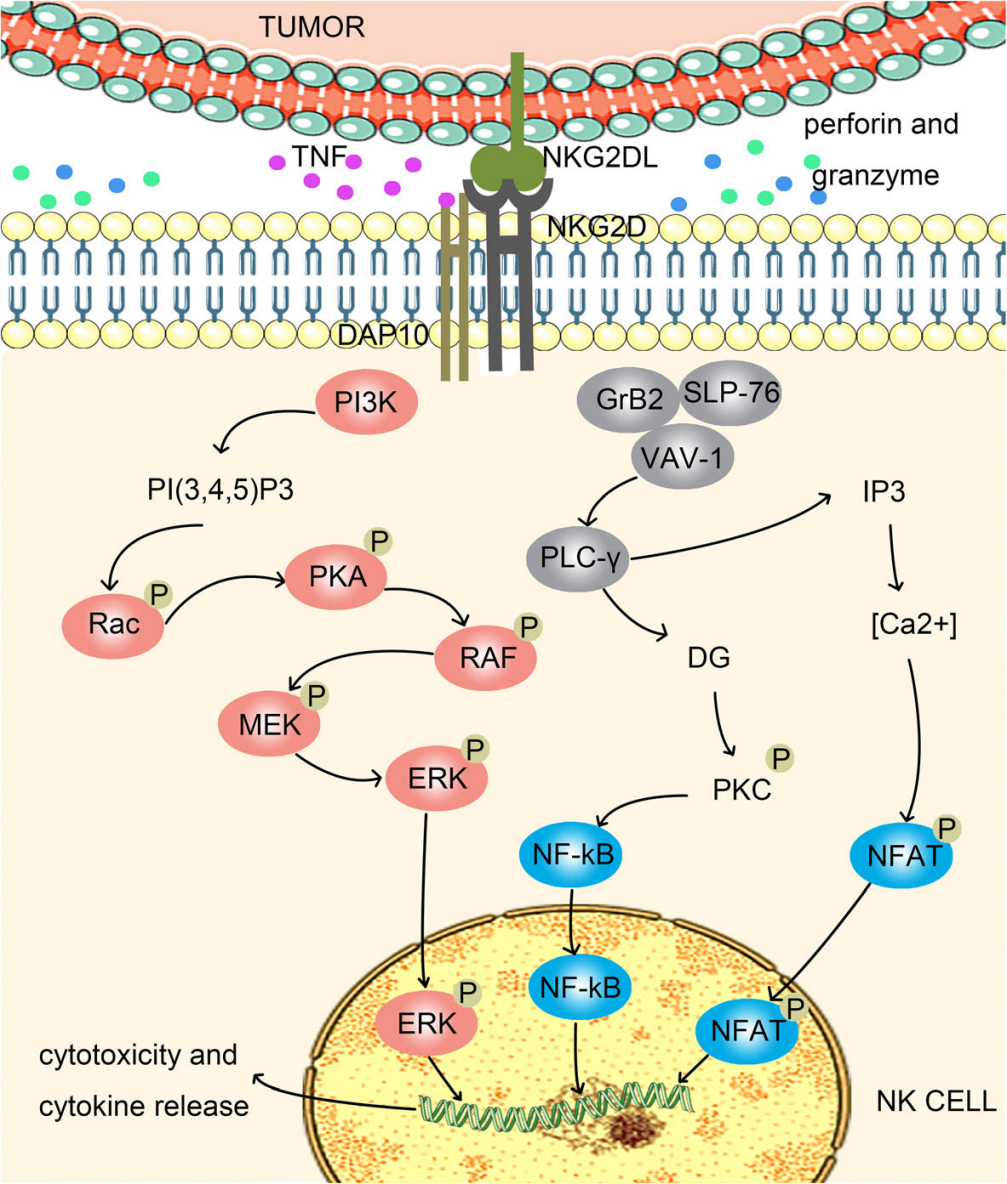
Function of NKG2D in NK cells. Humans only express one NKG2D subtype, NKG2D-L (long), which binds only to DAP10. DAP10 contains the YXXM motif, which recruits PI3K and GRB2, activates the Rac1/PAK/c-RAF/MEK/ERK and GrB2/VAV-1 pathways, and finally induces NK cells exerting cytotoxic effects, releasing cytokines, and killing tumor cells via perforin/granzymes, TNF-α/TNF-R1, and Fas/FasL

In NK cells, activation of PI3K produces the lipid product PI(3,4,5)P3 to activate Rac, thereby activating the Rac1/p21-activated kinase (PAK)/c-RAF/mitogen-activated protein kinase kinase (MEK)/extracellular signal-regulated kinase (ERK) pathway [18, 19]. In addition to the recruitment of PI3K, the NKG2D complex in human NK cells also recruits GRB2. Subsequently, the GRB2/Vav guanine nucleotide exchange factor 1 signaling pathway is activated, which leads to phospholipase C (PLC) activation. PLC activation finally activates the downstream IP3/Ca^2+^ and dendritic cell (DC)/protein kinase C pathways. Activation of the PI3K signaling pathway and the GRB2 signaling pathway leads to an increase in intracellular calcium concentration in NK cells, actin cytoskeleton rearrangement, and activation of transcription factors [20]. Recombination of the actin cytoskeleton ultimately leads to the formation of immunological synapses between tumor cells and NK cells. Secretion vesicles containing perforin/granzymes in NK cells release perforin, and granzymes induce tumor cell apoptosis by fusing with the membrane. Activation of transcription factors induces NK cells expressing and secreting various cytokines, including FasL, tumor necrosis factor (TNF), and TNF-related apoptosis-inducing ligand, which kills tumor cells via the Fas/FasL pathway and the TNFα/TNF-receptor 1 (TNF-R1) pathway (Fig. 1).

**Fig. 2:**
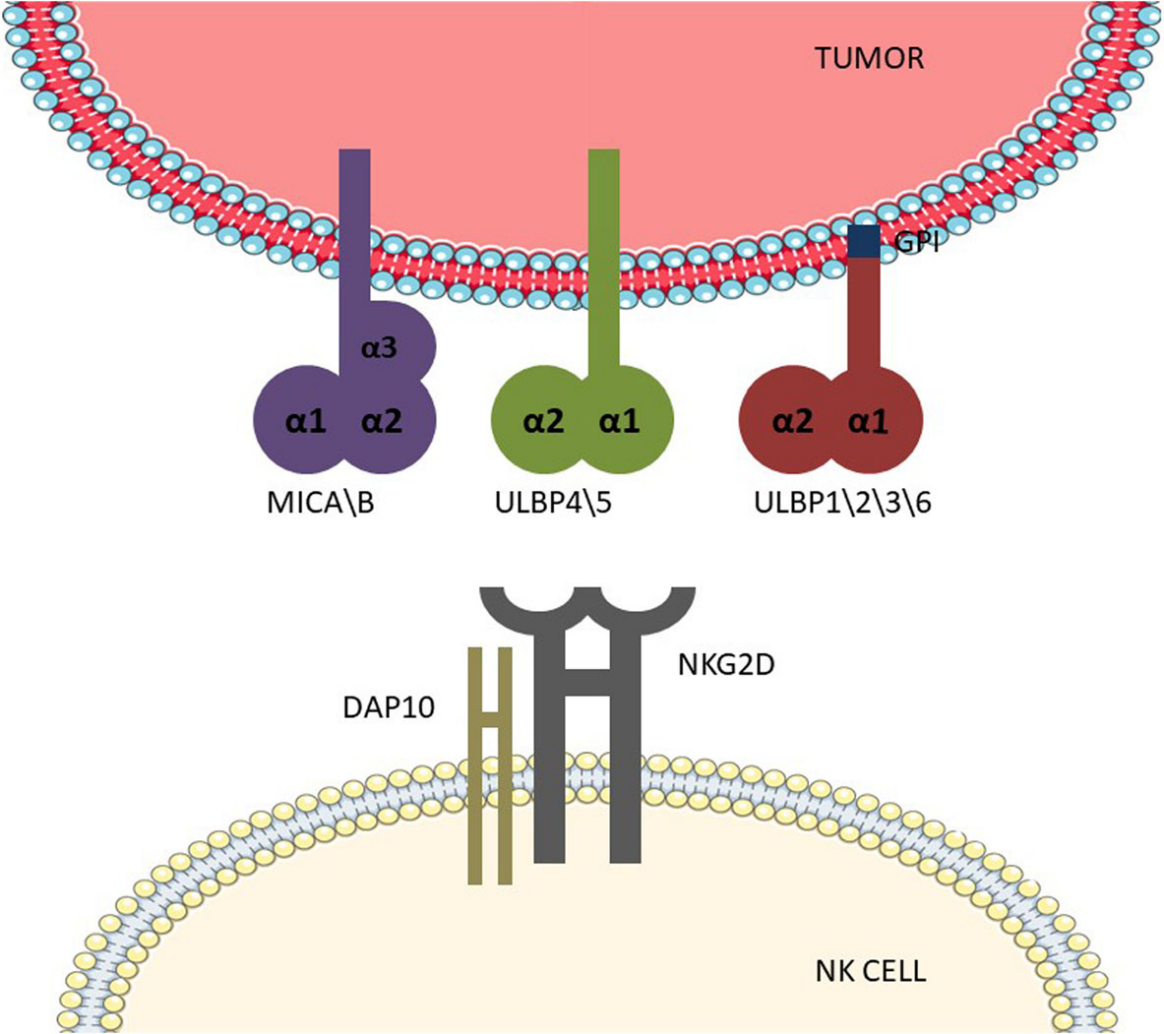
NKG2D receptor and its ligand. MICA\B has α1–3 domains, a transmembrane structure, and a cytoplasmic tail. ULBP1–6 have only the α1 and α2 domains; among these, ULBP1, − 2, − 3, and − 6 have a GPI-anchoring structure, and ULBP4 and − 5 have a transmembrane structure and a cytoplasmic tail. NKG2D is a homodimer of a type II transmembrane glycoprotein that binds noncovalently to DAP10 in the transmembrane domain

NKG2D recognizes a wide range of ligands. In humans, the NKG2D ligand (NKG2DL) includes MICA\B and UL16-binding proteins 1–6 (ULBP1–6), also known as retinoic acid early transcripts 1 [21]. NKG2DL is structurally similar to MHC class I molecules. MICA\B has the same α1, α2, and α3 domains as MHC class I, in which the α3 domain is an Ig-like domain, whereas ULBPs have only α1 and α2 domains. ULBP1, − 2, − 3, and − 6 are GPI anchoring receptors, and ULBP4 and − 5 have a transmembrane domain and cytoplasmic tail [22]. Unlike NKG2D receptor, NKG2DL is polymorphic. MICA has about 100 alleles, whereas MICB has 40 alleles. Different isomers affect the expression of MICA and MICB and the affinity with NKG2D to alter the effects of the NKG2D receptor\NKG2DL axis (Fig. 2), thus altering NK cell activity. NKG2DL is typically not expressed on normal cells and is only expressed when the cells are infected or undergo malignant transformation. NK cells can kill tumor cells without damaging other normal cells. Although the expression of MHC class I molecules in tumor cells is too low to induce the activation of cytotoxic T lymphocytes, the MHC class I molecule is a ligand for the major inhibitory receptor KIR on NK cells. Therefore, inhibition of NK cells by tumor cells is relieved, and the balance between inhibitory and active receptors is disrupted, which may active NK cells. Moreover, NKG2DL is expressed on tumor cells, and NK cells are activated after NKG2DL activates NKG2D, enabling clearance of tumor cells by NK cells. There are various mechanisms in the body that regulate the expression of NKG2D receptor and NKG2DL, thereby affecting clearance of tumor cells by the immune system and causing tumor escape.

### NKG2D-mediated tumor immune escape

Studies have confirmed that in various cancers, including cervical cancer, pancreatic cancer, and melanoma, NKG2D receptor expression is significantly decreased on immune cells, such as NK cells and CD8+ T cells, thereby promoting tumor immune escape.

Changes in NKG2D expression in NK cells may be regulated by a variety of factors, including changes in cellular activity factors and the physicochemical features of the tumor microenvironment (TME). The TME is composed of a variety of cells and molecules, including tumor-associated fibroblasts, tumor-associated macrophages, Tregs, immunoregulatory enzymes (e.g., arginase and cyclooxygenase-2 [COX-2]), and immunosuppressors (e.g., interleukin [IL]-10, transforming growth factor-β [TGF-β], vascular endothelial growth factor [VEGF], prostaglandin E_2_ [PGE_2_], and programmed death-ligand 1). Tumor cells and immunosuppressive cells express or secrete podocalyxin-like protein 1 (PCLP1), activin-a, indoleamine-pyrrole 2,3-dioxygenase (IDO), PGE_2_, TGF-β, and macrophage migration inhibitory factor (MIF) in the TME to mediate NKG2D downregulation.

PCLP1 belongs to the CD34 cell surface glycoprotein family. In breast malignancies, PCLP1 expression on tumor cells reduces the expression of NK cell-activated receptors, such as NKG2D, in a contact-dependent manner to resist NK cell-mediated cytolysis [23]. Pietra et al. found that IDO and PGE_2_ play major roles in the inhibition of NKG2D expression on NK cells in melanoma cells [24]. The IDO metabolite kynurenine inhibits IL-2-mediated expression of NKG2D receptor in NK cells via the c-Jun N-terminal kinase (JNK) pathway [25]. PGE_2_ blocks IL-15-mediated upregulation of NKG2D expression on NK cells [26]. PGE_2_ acts by binding to EP2 and EP4 receptors on the surface of NK cells, a class of G-protein coupled receptors [27]. EP2 and EP4 are coupled to Gs protein. PGE_2_ binds to EP2/4 and inhibits *NKG2D* transcription via the adenylate cyclase (AC)/cAMP/protein kinase A (PKA) pathway [28]. MIF is a tumor-derived protein that inhibits the activity of the transcription factor p53 and activates cyclin D1 and E2F transcription factors, which can activate the ERK1/2 and AKT pathways, stimulate tumor cell proliferation, enhance tumor cells migration and metastasis, and promote tumor angiogenesis [29]. Studies have found that MIF can downregulate the expression of NKG2D on the surface of NK cells and CD8+ T cells, permitting tumor cells to undergo immune escape from NK cells and CD8+ T cells [29–31]. TGF-β can downregulate NKG2D expression; however, the mechanisms are still unclear. Studies have shown that TGF-β downregulates NKG2D expression on NK cells by decreasing DAP10 expression at the transcriptional and translational levels [32].

Hypoxia is an important feature of the TME, which can directly or indirectly induce the secretion of immunosuppressive molecules, such that NK cells lose the ability to upregulate NKG2D expression through IL-2 and other cytokines [33]. Under hypoxic conditions, tumor cells can secrete a variety of chemokines to recruit immunosuppressive cells that secrete cytokines, such as TGF-β, thereby downregulating NKG2D expression. For example, in ovarian cancer, tumor cells secrete CCL28 to recruit Tregs [34]; hypoxia-inducible factor-1 (HIF-1) induced by hypoxia directly downregulates Foxp3 expression by combining with the *Foxp3* promoter region, inducing Treg formation [35], and regulates the function and differentiation of MDSCs [36]. Moreover, HIF-1 can upregulate COX-2 in tumor cells, and COX-2 can convert arachidonic acid to PGE_2_ [37]. Additionally, in tumor cells, hypoxia stress induces upregulation of the transcription factor NANOG, which can directly bind to the *TGF-β* promoter region and upregulate TGF-β expression [38] (Table 2).

**Table 2:** Molecules alter the expression of NKG2D on NK cells

Molecule	Mechanism	Function	Reference
PCLP1	Contact-dependent manner	↓	[23]
IDO	JNK pathway	↓	[24, 25]
PGE2	EP2/4, AC/cAMP/PKA pathway	↓	[26–28]
MIF	n.d	↓	[29–31]
TGF-β	n.d	↓	[32]
HIF-1	↑PGE2, TGF-β	↓	[37, 38]
IL-12	n.d	↑	[96]
IL-15	n.d	↑	[97]
IL-2	n.d	↑	[98]

*n.d* not determined, ↑: increase; ↓: decrease

**Fig. 3:**
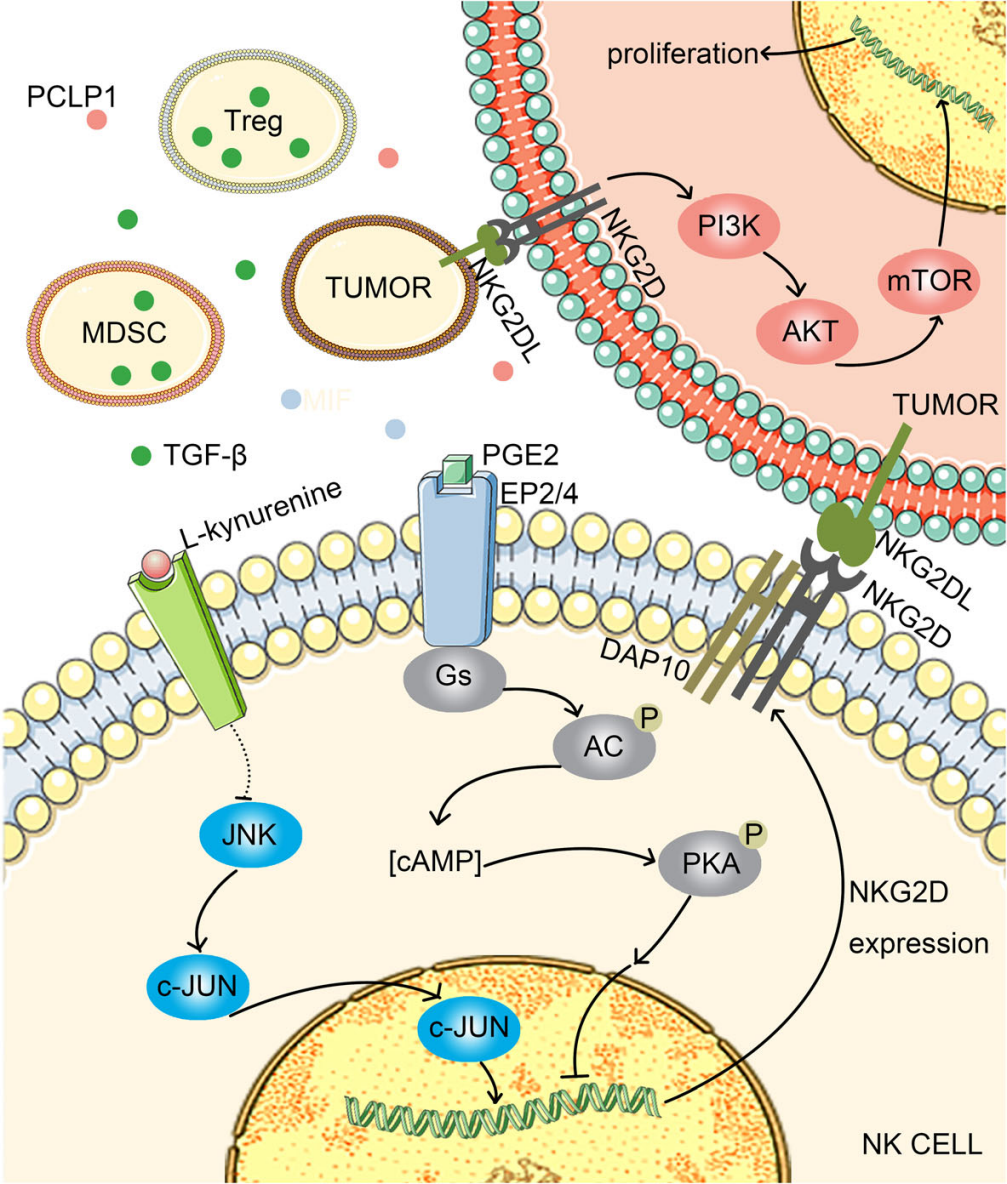
NKG2D-mediated tumor evasion. PGE_2_ binds to EP2/4 and inhibits *NKG2D* transcription via the AC/cAMP/PKA pathway. Kynurenine inhibits *NKG2D* transcription via the JNK pathway. Tregs and MDSCs secrete cytokines, such as TGF-β, and inhibit NKG2D expression. The tumor itself can also express NKG2D receptor, which binds to NKG2DL on adjacent tumor cells, activates the PI3K/AKT/mTOR signaling pathway, and promotes tumor proliferation

Recent studies have shown that tumor cells themselves can also express NKG2D receptor and compete with NK cells for binding to NKG2DL, thereby indirectly inhibiting the activation of NK cells. Downregulation of NKG2D receptor expression in tumor cells can increase the expression of cyclin E and cyclin-dependent kinase 2, reduce the expression of p21, and increase the number of tumor cells in G_1_ and S phases, leading to inhibition of tumor cell proliferation. At the same time, NKG2D receptor expression on tumor cells binding to NKG2DL expressed by adjacent tumor cells leads to autostimulation of the PI3K/AKT/mammalian target of rapamycin (mTOR) oncogenic signaling pathway, promotes tumor angiogenesis and metastasis, and enhances tumor proliferation (Fig. 3) [39].

### NKG2DL-mediated tumor immune escape

**Fig. 4:**
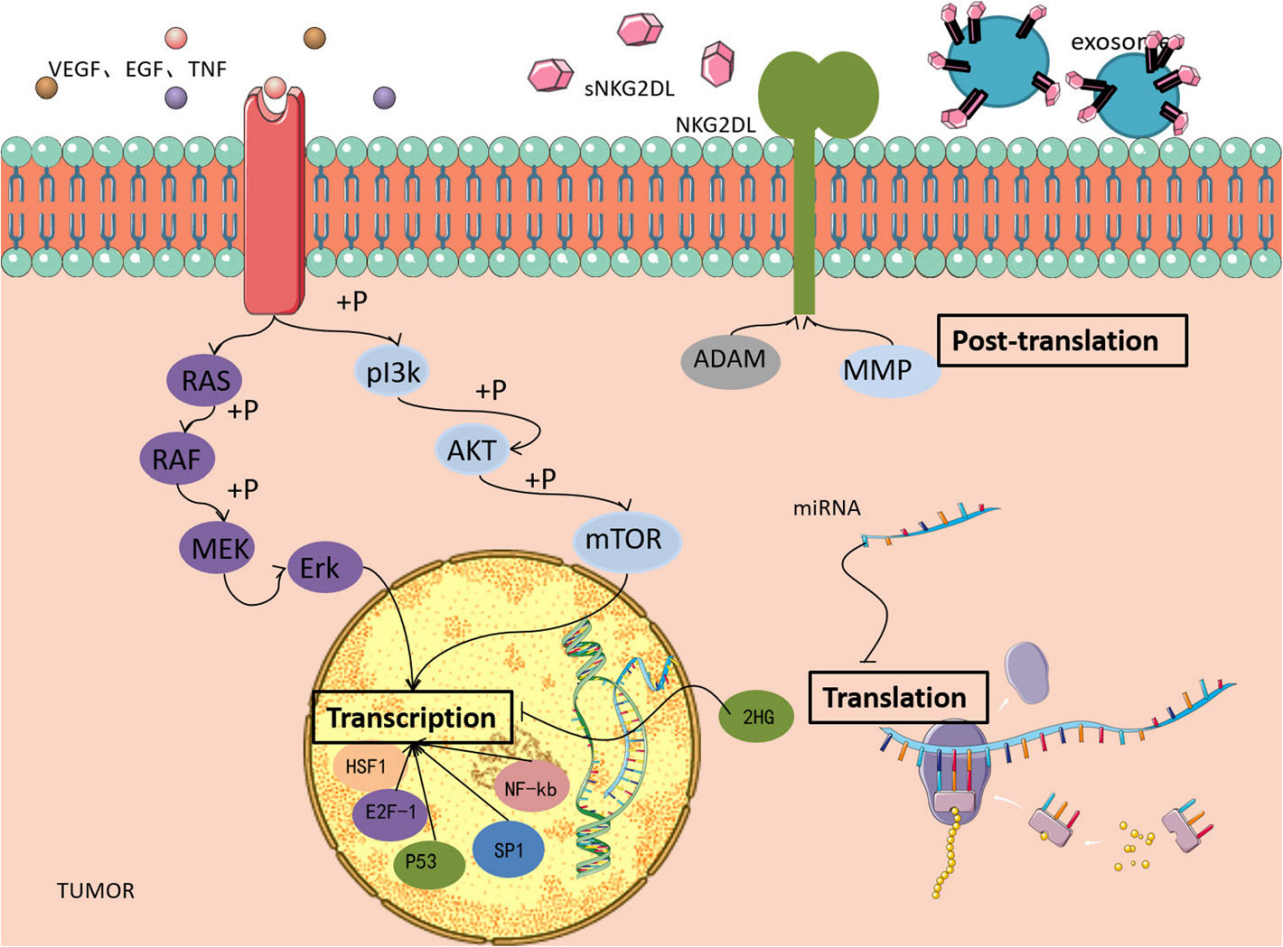
Levels of regulation of NKG2DL expression. Transcriptional level: 1) The transcription factors p53, E2F-1, HSF-1, and NF-κB promote the transcription of *NKG2DL* genes; 2) After acting on tumor cell membrane surface receptors, growth factors promote *NKG2DL* gene transcription through the PI3K/AKT pathway; 3) Expression of 2-HG due to *IDH* gene mutation can inhibit the transcription of the *NKG2DL* gene. Translational level: miRNA binding to *NKG2DL* mRNA affects its stability and decreases NKG2DL expression. Post-translational level: 1) ADAM and MMP hydrolyze the intracellular part of NKG2DL such that NKG2DL is secreted and the expression level is reduced; 2) The secretion of exosomes with NKG2DL can reduce the expression levels of NKG2DL and NKG2D receptor

Human NKG2DL includes MHC class I molecule-related proteins (MICA/B) and ULBP1–6. Malignant cells use a variety of mechanisms to reduce the levels of NKG2DL expression and escape NKG2D-mediated immune surveillance (Fig. 4).

#### Transcription-level regulation of NKG2DL expression

DNA damage induction and growth factor stimulation in the TME can activate various signaling pathways and upregulate the expression of NKG2DL at the transcriptional level. The reactive oxygen species (ROS)-induced DNA damage response promotes tumor immunity by binding the transcription factor E2F1 to the E2F1 binding site adjacent to the transcription initiation site of the *MICA* promoter [40]. Induction of the transcription factor p53 can strongly induce the transcription of *ULBP1* and *ULBP2* and then upregulate their expression on the tumor cell surface. In the ATM/ATR signaling pathway, downstream p53 is activated and binds to the p53 response element on the *ULBP1*/*ULBP2* promoter to upregulate the transcript levels of *ULBP1*/*ULBP2* [41]. The TME is rich in growth factors, such as VEGF and epidermal growth factor, which bind to their receptors and then activate the PI3K/AKT and RAS/RAF pathways. After the PI3K/AKT pathway is activated, the downstream mTOR complex 1 (mTORC1) is activated immediately. Phosphorylated ribosomal kinase activates ribosomal p70S6 kinase protein, which activates the ribosomal 40S protein S6 and eventually promotes the translation of 5′-terminal mRNA and the expression of NKG2DL. In contrast, mTORC1 activates and phosphorylates initiating factor 4E binding protein 1, initiates translation, encodes cell cycle regulatory proteins, and promotes tumor cell proliferation [42]. However, at the transcriptional level, the expression of *NKG2DL* can also be downregulated, leading to tumor immune escape. Aberrant methylation of NKG2DL-encoding DNA or low acetylation of histones causes NKG2DL silencing [43, 44]. Mutations in the isocitrate dehydrogenase (*IDH*) gene cause loss of the original enzyme activity and transform alpha-ketoglutarate into a hydrogenase inhibitor, 2-hydroxyglutaric acid (2-HG), which induces epigenetic and metabolic reprogramming and silences ULBP1/ULBP3 [45]. In addition, the TME contains abundant cytokines, among which interferon IFN-γ can downregulate the expression of NKG2DLH60 in 3′-methylcholangiosarcoma [46]. In melanoma, IFN-γ triggers signal transducer and activator of transcription-1 signaling, which lowers the expression of *MICA* mRNA and blocks MICA expression in tumor cells. IFN-γ not only regulates *MICA* transcript levels but also promotes the hydrolysis of MICA by matrix metalloproteinases at the post-translational level [47]. TGF-β inhibits the transcription of *MICA*, *ULBP2*, and *ULBP4* mRNA, whereas *MICB*, *ULBP1*, and *ULBP3* mRNA levels remained unchanged. TGF-β was upregulated in malignant glioma and selectively inhibited the expression of MICA and ULBP2/4 but had no significant effect on MICB and ULBP1/3 [48]. Among the transcription factors heat shock factor 1 (HSF1), specificity protein 1 (SP1), and nuclear factor (NF)-κB, HSF1 regulates the expression of MICA/B. SP1 binds to the promoter of *MICA/B* and participates in transcription regulation. Knockout of these transcription factors results in downregulation of MICA/B [49]. Endothelial cells treated with TNF-α exhibit induction of NF-κB binding to the promoter of *MICA*, resulting in upregulation of *MICA* expression [50].

#### Translation-level regulation of NKG2DL expression

The regulation of translation occurs mainly through microRNAs (miRNAs), noncoding small RNA molecules. MiRNAs, such as *miR-20a*, *miR-93*, *miR-106*, and *miR-10b*, can bind with the 3′-untranslated region (UTR) of the *NKG2DL* promoter to inhibit the translation or damage the stability of mRNA [51], resulting in inhibition of NKG2DL expression at the translational level [52, 53]. The forced expression of *miR-889* prevents MICB upregulation, and *miR-34* causes downregulation of ULBP2 [54]. In glioma cells, miRNA can downregulate the expression of NKG2DL. Additionally, combination of the hypoxic characteristics of glioblastoma and the effects of miRNAs can enable tumor cells to evade NKG2D-mediated immune surveillance. In colon and prostate cancer cells and melanoma, miRNAs regulate the degradation of specific mRNAs, which are then involved in NKG2DL regulation [53, 55, 56].

#### Post-translational-level regulation of NKG2DL expression

After translation, the expression of NKG2DL is still affected by proteolytic enzymes, shedding, secretion, and other factors. NKG2DL can be degraded by some proteolytic enzymes, such as RAZTI1, which degrades ULBP2. Systemic downregulation of NKG2D on NK cells in patients with cancer has been observed in many studies and is attributable to soluble NKG2DL (sNKG2DL) [57]. Tumor cells reduce NKG2DL surface expression through proteolysis, resulting in sNKG2DL release [58]. Palmitoylation of two cysteine residues in the MICA tail is required during the process of NKG2DL shedding [59]. NKG2DL can be hydrolyzed by some integrins and metalloproteinases, including ADAM9, ADAM10, ADAM17, matrix metalloproteinase (MMP) 9, MMP14, and the disulfide isomerase Erp5 to form soluble NKG2DL [60]. In human glioblastoma cell lines, only ULBP2 is released in the soluble form through the proteolytic activities of ADAM10 and ADAM17 [61]. MMP9 is associated with myeloma, inhibits the release of sMICA, and promotes immune escape. Moreover, MMP2 mediates the shedding of MICA in renal carcinoma cell membranes [62]. MIC is the main NKG2DL, and the combination of exfoliated sMIC and NKG2D will lead to endocytosis of NKG2D receptor and its degradation by lysosomes, thus disrupting the tumor immune surveillance function of NKG2D [63]. Exfoliated NKG2DL affects the recognition and migration of NK cells, reduces the toxicity of NK cells, interferes with the maintenance of NK cell homeostasis, affects NK cell depletion, inhibits the immune surveillance function of NK cells, and initiates immune escape [64, 65]. In these previous studies, relevant shedding was found in prolactin tumors, mouse models, oral squamous cell carcinoma, breast cancer, prostate cancer, lung cancer, ovarian cancer, colon cancer, and other tumor-related diseases.

The level of shedding is regulated by a variety of factors, and IL-1β in the TME activates ADAM9 in human liver cancer cells to increase the production of sMICA [66]. Hypoxia increases the release of MIC, whereas the activation of nitric oxide signaling inhibits the release of MIC. The expression levels of Erp5 and GRP78 are correlated with the expression levels of membrane-bound MICA in patients with chronic lymphocytic leukemia. MIC harbors many polymorphisms; MICA has 100 alleles, and MICB has 40 alleles. The conformational changes caused by MICA polymorphisms affect the accessibility of ADAM proteases or molecular chaperones. For example, mica-129met isomers are more likely to fall off and form sMICA than mica-129 val isomers [51]. Moreover, the different affinities of MICA alleles may affect the threshold of NK cell stimulation and T-cell regulation. Cervical cancer is one of the earliest malignant diseases found to be associated with MICA polymorphisms. Because patients with cervical cancer carrying the A5.1 allele have less membrane-bound MICA, this may impair their ability to alert the immune system to human papillomavirus infection or tumor changes and increase the risk of disease [67].

In some tumors, NKG2DL is secreted in exosomes, thereby reducing cell surface expression. For example, in PC-3 prostate cancer cells, MICA is secreted by exosomes and exits the tumor cells. Although NKG2DL expression on the surface of tumor cells is decreased significantly, thereby contributing to immune escape, soluble NKG2DL (snkg2dl) binds to NKG2D receptor and triggers its internalization ability [68]. Studies have shown that the expression of TGF-β and NKG2DL (MICA/B, ULBP1–3) on the exosome surface in the extrapleural fluid of malignant mesothelioma downregulates the expression of NKG2D on immune cells, which is also an important factor causing tumor immune escape [68].

#### Regulation mechanism of NKG2DL expression in various tumors

Different tumors have their own mechanisms for escape, and the same pathways may be involved. There are three levels in NKG2DL related pathways. At the transcriptional level, IFN-γ inhibits transcription of *NKG2DL* mRNA. At the translation level, miRNA and siRNA downregulate the expression of *NKG2DL* mRNA. At the post-translational level, exosomes play a vital role (Table 3).

**Table 3:** Regulation mechanisms of NKG2DL expression in various tumors

Tumor type	Regulation level	Regulation mechanism	Reference
3′-Methyl cholangiosarcoma	Transcription level	IFN-γ downregulates the expression of NKG2DLH60	[46]
Melanoma	Transcription level	IFN-γ triggers signal transducer and activator of transcription-1 signaling, lowers *MICA* mRNA expression, and blocks MICA expression in tumor cells	[47]
Malignant glioma	Transcription level	TGF-β was upregulated and selectively inhibited the expression of MICA and ULBP2/4	[48]
Colon and prostate cancer and melanoma	Translation-level	miRNAs regulate the degradation of specific mRNAs, which are then involved in NKG2DL regulation	[53, 55, 56]
Human glioblastoma	Post-translational level	ULBP2 is released in the soluble form through the proteolytic activities of ADAM10 and ADAM17	[61]
Malignant mesothelioma	Post-translational level	TGF-β and NKG2DL (MICA/B, ULBP1–3) on the exosome surface downregulate the expression of NKG2D on immune cells	[68]

#### Effects of viruses on NKG2DL expression

Studies have shown that the development of some tumors is associated with certain viruses, and the risk of related tumors increases sharply due to multiple viral infections. These viruses affect the expression of NKG2DL in infected deteriorating cells via different pathways in order to induce immune escape in deteriorating cells.

Moreover, the human cytomegalovirus (HCMV) glycoproteins UL16 and ULl42 downregulate the expression of NKG2DLs [69], and targeting of MICB mRNA by the viral miRNA HCMV-miR-UL112 can reduce MICB translation [70]. Similarly, two polyomaviruses, John Cunningham virus and BK virus,can use the same miRNAs to silence the expression of ULBP3 and cause immune evasion [71]. Hepatitis B virus (HBV) releases HBc and HBx proteins to promote the expression of transcription factors GATA-2 and GATA-3 after infection, and GATA-2 and GATA-3 specifically inhibit MICA/B expression by directly binding to the promoter region of MICA/B [72]. The hepatitis C virus protein NS3/4A is associated with immune invasion and leads to decreased NKG2D ligand expression through unexplored mechanisms [73]. These mechanisms inhibit the cytotoxic effects of NK cells via the downregulation of NKG2DL expression to achieve immune escape.

Second, viruses can also generate immune evasion by inhibiting the expression of NKG2DL on the surface of infected cells. Studies have shown that when cells are infected with wild-type adenovirus, the early adenovirus gene product E3/19 K protein increases the synthesis of the NKG2DLs MICA and MICB, and these products are then stored in the immature form in the endoplasmic reticulum. However, their expression on the cell surface is inhibited [74]. Similarly, vesicular stomatitis virus-infected cells will be strongly induced to express MICA mRNA; however, subsequent NKG2DL expression on the surface of the target cells is actively inhibited, although the mechanism had not yet been explored [75].

Human immunodeficiency virus (HIV)-1 infected cells achieve immune evasion by releasing soluble ligands (sNKG2DLs), leading to a decrease in NKG2DL on the cell surface and inhibition of NK cell function by sNKG2DL [76].

**Fig. 5:**
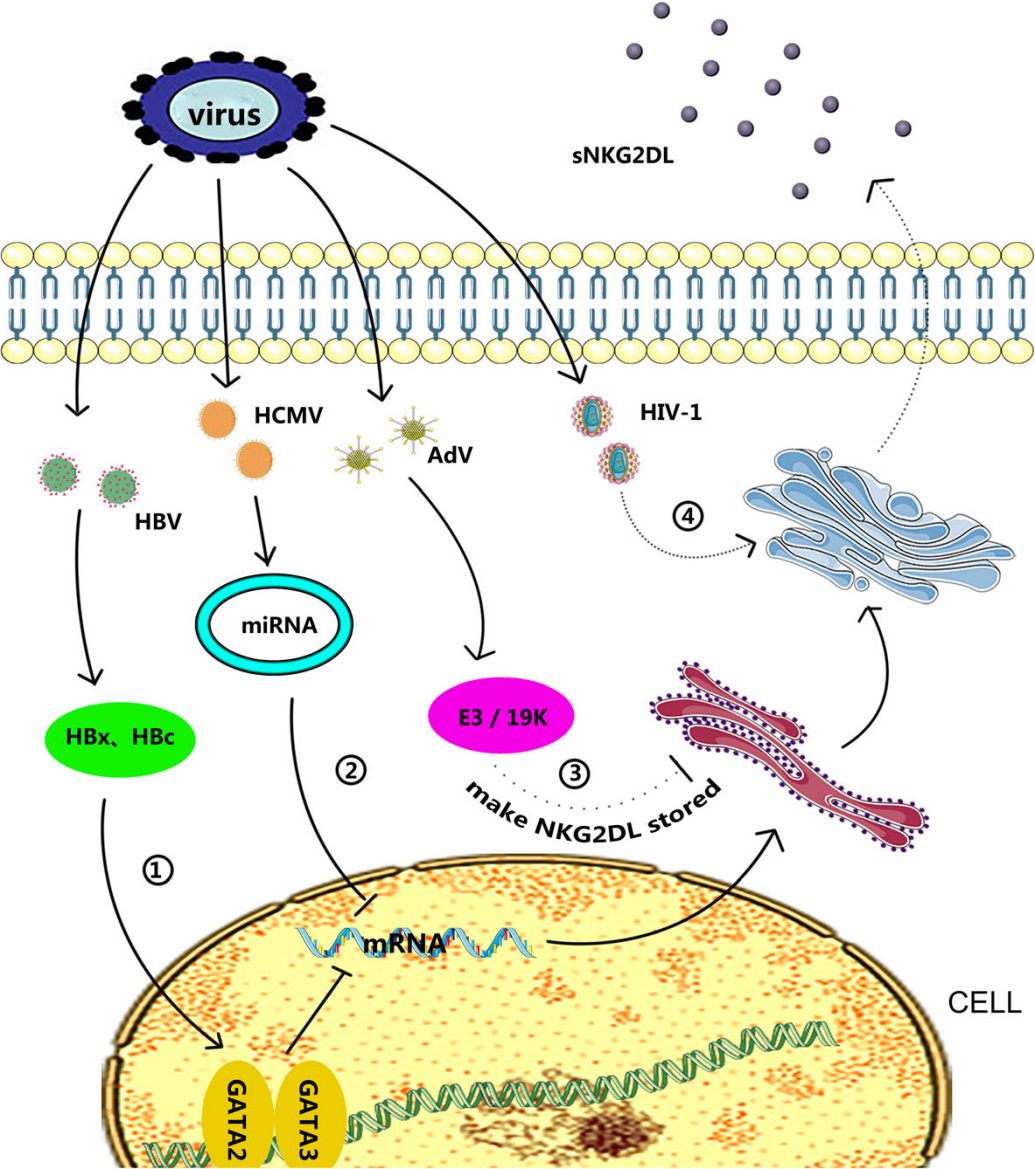
Effects of virus on NKG2DL expression. 1) HBV infection promoted the expression of GATA-2 and GATA-3 and directly bound to the promoter region of MICA/B to inhibit MICA/B expression. 2) Viral miRNA targeting mRNA reduced MICB translation. 3) E3/19 K protein increased the synthesis of the NKG2D ligands MICA and MICB and stored them in the immature form in the endoplasmic reticulum but inhibited their expression on the cell surface. 4) HIV-1 caused infected cells to release soluble ligands (sNKG2DL)

A variety of viruses have been shown to downregulate the expression of NKG2DL on the surface of infected cells. However, further studies are needed to elucidate the exact mechanisms. Whether some tumorigenic viruses also achieve immune escape by downregulating the expression of NKG2DLs remains unclear. (Table 4, Fig. 5).

**Table 4:** Effects of virus on NKG2D expression

virus	Mechanism	Function	Reference
HCMV	HCMV-miR-UL112 interferes with *MICB* mRNA	MICB↓	[70]
JCV, BKV	JCV/BKV miR-J1/B1-3p interferes with *ULBP3* mRNA	ULBP3↓	[71]
HBV	HBc and HBx promote the expression of GATA-2 and GATA-3	MICA/B↓	[72]
HCV	NS3/4A protein	NKG2DL↓	[73]
AdV	E3/19 K protein	NKG2DL stored	[74]
VSV	n.d	MEI	[75]
HIV	n.d	sNKG2DLs↑	[76]

*n.d* not determined, *MEI* membrane expression inhibition, ↓: decrease; ↑: increase

#### Effects of hormones on the expression of NKG2DL

Studies of the factors affecting NKG2D receptor/NKG2DL expression have shown that some hormones play negative roles in the immune escape of tumor cells.

Vasoactive intestinal peptide (VIP) has been shown to enhance Th2 cell responses, inhibit Th1 cell proliferation [77, 78], affect B-cell differentiation [79, 80], and inhibit NK cell activity. Therefore, VIP is considered an important immunosuppressive neuropeptide. Wang et al. [81] showed that VIP can inhibit the cytotoxicity of NK cells to MKN45 gastric adenocarcinoma cells and inhibit the expression of NKG2D receptor, DAP10, and NF-κB in NK cells; gastric cancer cells may evade immune clearance via this mechanism. Thus, VIP antagonists may be helpful for the study of anticancer drugs in patients with gastric cancer.

Estrogen and estrogen signaling pathways can induce the epithelial-mesenchymal transformation and play key roles in the occurrence and pathogenesis of lung cancer. Estrogen can help lung cancer cells escape NKG2D-mediated immune surveillance, and *MICA/B* mRNA and secreted protein levels are upregulated by estradiol in lung adenocarcinoma cell lines via a mechanism mediated by enhanced expression of ADAM17 [82]. Secretion of MICA/B in lung adenocarcinoma cell lines will downregulate NKG2D receptors on the surface of NK cells and impair the cytotoxic activity of NK cells [83], enabling lung cancer cells to escape NKG2D-mediated immune surveillance.

Intracellular ULBP2 synthesis can be significantly increased by treatment with adrenocorticotropic corticotropin-releasing hormone in HeLa cervical cancer cells and is released from the cells by metalloproteinase to become soluble ULBP2. This protein can reduce NK cell-mediated tumor killing effects.

Some studies have evaluated the effects of hormones on the expression of NKG2D receptor\NKG2DL. As an important part of the human homeostasis regulation network, hormones can be further studied to determine their effects on the TME and their relationship with tumor immune evasion. Additionally, studies of the effects of hormones on tumor immune evasion will be helpful for the treatment of corresponding tumors and the development of antitumor drugs.

### NKG2D receptor and NKG2DL are critical targets for cancer immune therapy

In recent decades, studies of the correlations among tumor immune escape, NKG2D receptor, and NKG2DLs have established a new direction in tumor treatment. Thus, maximizing the activation of NKG2D expression on immune cells and fully inducing the expression of NKG2DL in tumor cells have become core concepts in cancer research.

The effects of immunotherapy can be achieved by improving the expression of NKG2D on immune cells. Studies have shown that a variety of cytokines, including IL-2 and IL-12, can stimulate the expression of NKG2D on the cell surface, making this protein a focus in antitumor therapy. The induction of NKG2D expression by NK cells has been demonstrated in many trials and is widely used in antitumor therapy.

Notably, NK cells in patients with acute myeloid leukemia (AML) have a higher ability to activate receptors under IL-15 stimulation in vitro and produce higher levels of cytotoxicity against autologous AML cells. These preclinical data have provided important evidence for the use of IL-15 in the treatment of AML [84] and have suggested that IL-15 may also be effective for the treatment of metastatic melanoma [85]. ILs with high structural homology to IL-15, such as IL-21, may also have therapeutic potential. Mononuclear and multinuclear macrophages in the TME can generate ROS, which can mediate downregulation of NKG2D expression, whereas histamine can block this pathway and indirectly increase NKG2D expression. Based on this, IL-2 induces the killing of human AML cells in vitro. The combination use of histamine and IL-2 can protect patients with AML from leukemia recurrence [86]. TGF-β is also an important component in the TME that not only promotes the occurrence and growth of tumor cells but also has immunosuppressive activity, particularly by downregulating the expression of NKG2D on the surface of NK cells. In this regard, anti-TGF-β monoclonal antibodies and antisense oligonucleotides can be used to reduce TGF-β expression and achieve therapeutic effects [87].

Upregulation of NKG2DL expression on tumor cells can also achieve therapeutic effects. Romidepsin and Vorinostat have been approved by the US Food and Drug Administration for the treatment of cutaneous T-cell lymphoma, both of which increase NKG2DL expression. Gefitinib can upregulate the expression of ULBP1, ULBP2, and MICA in tumor cells and can promote the expression of NKG2D in NK cells, thereby inhibiting tumor escape [6].

The dual effects of anticancer drugs have always hindered the clinical use of these drugs. Therefore, the focus of current research is maximization of the beneficial effects of these drugs. Bortezomib plays an important role in promoting the cytotoxicity of NK cells in anticancer therapy and shows enhanced effects after large consumption of Tregs [88]; however, studies have shown that bortezomib can weaken this effect by resisting tumor-specific T cells [89, 90]. In addition, in vitro experiments have demonstrated that bortezomib has pro-apoptotic effects on NK cells and induces downregulation of NKp46 expression [91]. b-AP15 was found to overcome the harmful effects of bortezomib on T cells [89]. Targeted therapy with a histone deacetylase inhibitor (HDACi) can not only enhance the expression of MICA/B but also upregulate the expression of NKG2D on innate immune cells to enhance their sensitivity, which finally improves the tumor killing effects of cytokine-induced killer cells with minimal effects on normal cells. Nevertheless, after treatments with first- and second-generation HDACis (e.g., vorinostat, trichostatin A, valproic acid, and apicidin) on various cancer cell lines, negative results were obtained by downregulating the expression of other NK cell-activating receptors (e.g., Bp-H6 and NKp30) and impairing NK cell recognition of tumor cells [92].

With the development of novel research models, experiments have gradually shifted from traditional chemotherapy methods, which aim to kill cells, to immunotherapy, which aims to reduce the side effects of traditional methods. HDACi target cell therapy can cause MICA/B shedding to increase; thus, pheophorbide A oxygenase (PAO) or matrix metalloproteinases inhibitors (MMPi) can be used for pretreatment. The latter is more suitable for clinical use based on its efficacy and toxicity [85]. As additional examples, trastuzumab and cetuximab are directed against epithelial tumor cells from breast or colon adenocarcinoma, respectively.

Based on the results of CAR-T cell therapy in hematological malignancies, CAR-NK cell therapy has also shown efficacy in the treatment of solid tumors.NK-92 cells can be modified to express CAR protein against various cancer targets, including CD20 against lymphoma and leukemia, CD19 against chronic lymphocytic leukemia (CLL), and neuroblastoma GD2, and EpCAM for breast cancer [93] One study showed that EpCAM-specific CAR-NK-92 cells exhibited antitumor effects in vitro through cytotoxicity and release of cytokines, including IFN-γ, perforin, and granzyme B. The antitumor effects of CAR-NK-92 cells in a colorectal cancer (CRC) mouse model could be enhanced by combination with regorafenib [94].

In vitro NK cell expansion has resulted in breakthroughs in antitumor therapy research. Owing to the downregulation of NK cell receptors in the tumor microenvironment, NK cell immune function is restricted, and the NK cell system and cell metastasis can be expanded in vitro to increase the number of high-function NK cells in the body, thereby enhancing the antitumor effects of the NK cell system. However, it is unlikely that the disease will be completely eradicated via this pathway because even after extensive treatment, the tumor will recur after the disease-free period. Therefore, expanded NK cells can be combined with immunotherapeutic strategies, such as CAR-T, CAR-NK, and anti-PD-1/PD-L1 targeting, as well as chemotherapy, radiotherapy and tumor virus treatment strategies to achieve tumor elimination [95].

## Conclusion and perspectives

Various components in the TME can regulate the expression of NKG2D receptor and NKG2DL through different mechanisms. Deviation of the NKG2D receptor/NKG2DL ratio from normal values can facilitate escape of tumors from NK cell-mediated immune surveillance. Based on this tumor immune escape pathway, the use of medical means to control factors affecting the expression of NKG2D receptor and NKG2DL is being investigated as an alternative treatment for tumors.

Identification of pathways of NKG2D-mediated immune escape can facilitate the development of new drugs for cancer treatment, such as drugs that can degrade metalloproteinases (ADAM9, ADAM10, ADAM17, MMP9, MMP14, and Erp5) to reduce the production of sNKG2DL.

Combining new technologies to enhance the cytotoxicity of NK cells on tumor cells is also worth studying. For example, recombinant protein technology may be used to explore and identify recombinant proteins having the dual ability to recognize NKG2D and tumor cells, becoming a bridge connecting immune cells and tumor cells and enhancing the immune surveillance function of NK cells.

However, many factors related to NKG2D and tumor escape are still unclear. For example, more studies are still needed to elucidate the mechanisms regulating the effects of certain viruses on the expression of NKG2DL on tumor cells; the mechanisms regulating hormones on immune cells, tumor cells, and components in the TME, which may have direct or indirect effects on future tumor treatment based on the wide range of targets of hormones; and the mechanisms through which tumor cells express NKG2D.

## Abbreviations

4E-BP1: Initiating factor 4E binding protein 1
alpha-KG: Alpha-ketoglutarate
CIK: Cytokine Induced Killer cells
CLL: Chronic lymphocytic leukemia
COX-2: Cyclooxygenase-2
CRC: colorectal cancer
DAP10: DNAX activating protein 10
DAP12: DNAX activating protein 12
DDR: DNA damage response
EGF: Epidermal growth factor
HCC: Human liver cancer cells
HDACi: Histone deacetylase inhibitor
HIF-1: Hypoxia-inducible factor-1
IDH: Isocitrate dehydrogenase
IL-10: Interleukin-10
ITAM: Immunoreceptor tyrosine-based activation motif
ITIM: Immunoreceptor tyrosine-based inhibitory motif
KIR: Killer immunoglobulin-like receptor
KLR: Killer lectin-like receptor
MCA: Methylcholangiosarcoma
MDSC: Myeloid-derived suppressor cells
MICA/B: MHC class I molecule related proteins
MIF: Migration inhibitory factor
MMPi: Matrix metalloproteinase inhibitor
NCR: Natural cytotoxicity receptor
NK: Natural killer
NKC: NK gene complex
NKG2D: Natural killer group 2 member D
NKG2DL: NKG2D ligand
P53RE: P53 response element
PAO: Pheophorbide a oxygenase
PCLP1: Podocalyxin-like protein 1
PD-L1: Programmed death-ligand 1
PGE2: Prostaglandin E2
RAET1: Retinoic acid early transcripts 1
ROS: Reactive oxygen species
sNKG2DL: Soluble NKG2DL
TGF-β: Transforming growth factor-β
TME: Tumor microenvironment
ULBP1–6: UL16-binding proteins
VEGF: Vascular endothelial growth factor
VIP: Vasoactive intestinal peptide
